# The New Occurrence of Antiphospholipid Syndrome in Severe COVID-19 Cases with Pneumonia and Vascular Thrombosis Could Explain the Post-COVID Syndrome

**DOI:** 10.3390/biomedicines13020516

**Published:** 2025-02-19

**Authors:** Mirjana Zlatković-Švenda, Melanija Rašić, Milica Ovuka, Slavica Pavlov-Dolijanović, Marija Atanasković Popović, Manca Ogrič, Polona Žigon, Snežna Sodin-Šemrl, Marija Zdravković, Goran Radunović

**Affiliations:** 1 Institute of Rheumatology, 11000 Belgrade, Serbia; rasicmelanija@gmail.com (M.R.); slavicapavlovdolijanovic@gmail.com (S.P.-D.); atanaskovic.mara@gmail.com (M.A.P.); g.radunovic47@gmail.com (G.R.); 2 Faculty of Medicine, University of Belgrade, 11000 Belgrade, Serbia; 3 Faculty of Medicine Foča, University of East Sarajevo, 73300 Foča, Bosnia and Herzegovina; 4 Institute for Cardiovascular Diseases Dedinje, 11040 Belgrade, Serbia; movuka98@gmail.com; 5 Clinical Hospital Center Pancevo, 26000 Pancevo, Serbia; 6 Department of Rheumatology, University Medical Centre Ljubljana, 1000 Ljubljana, Slovenia; manca.ogric@kclj.si (M.O.); polona.zigon@kclj.si (P.Ž.); 7 FAMNIT, University of Primorska, 6000 Koper, Slovenia; ssodin1@yahoo.com; 8 Clinical Hospital Center Bežanijska kosa, 11071 Belgrade, Serbia; sekcija.kardioloska@gmail.com

**Keywords:** antiphospholipid syndrome, immunology, COVID-19, SARS-CoV2, post-COVID syndrome, vascular thrombosis, antiphospholipid antibodies (aPLs), anticardiolipin (aCL) antibodies, anti-β2-glycoprotein I (anti-β2GPI) antibodies, anti-phosphatidylserine-prothrombin (aPS/PT) antibodies

## Abstract

**Introduction:** The classification of antiphospholipid syndrome (APS) comprises clinical criteria (vascular thrombosis or obstetric complications throughout life) and laboratory criteria (antiphospholipid antibodies (aPLs) positivity, confirmed at least twice at 12-week interval). **Methods:** In 100 patients admitted to the hospital with COVID-19 pneumonia, thrombosis and pregnancy complications were recorded during the hospital stay and in personal medical history. They were tested for nine types of aPLs at four time points (admission, deterioration, discharge, and 3-month follow-up): anticardiolipin (aCL), anti-β2-glycoproteinI (anti-β2GPI), and antiphosphatidylserine/prothrombin (aPS/PT) isotypes IgM/IgG/IgA. **Results:** During hospitalization, aPLs were detected at least once in 51% of patients. All 7% of deceased patients tested negative for aPLs upon admission, and only one patient became aCL IgG positive as his condition worsened. In 83.3% of patients, intrahospital thrombosis was not related to aPLs. One patient with pulmonary artery and cerebral artery thrombosis was given an APS diagnosis (triple aPLs positivity on admission, double on follow-up). Personal anamnesis (PA) for thromboembolism was verified in 10 patients, all of whom tested negative for aPLs at admission; however, transition to aPLs positivity at discharge (as the disease subsided) was seen in 60% of patients: three of six with arterial thrombosis (at follow-up, two did not appear, and one was negativized) and three of four with deep vein thrombosis (one was confirmed at follow-up and diagnosed with APS, one was negativized, and one did not appear). At admission, the majority of the aPLs were of the aCL IgG class (58.8%). Unexpectedly, as the COVID-19 disease decreased, anti-β2GPI IgG antibodies (linked with thromboses) became newly positive at discharge (14.9%), as confirmed at follow-up (20.8%). **Conclusion:** The incidence of APS in our cohort was 2.0%, whereas in the general population, it ranges from 0.001% to 0.002%. The incidence might have increased even more if the four aPLs-positive patients with intrahospital thrombosis/history of thrombosis had attended follow-up. **Recommendation:** All patients with severe COVID-19 or post-COVID syndrome should be evaluated for current/previous thrombosis and tested for aPLs at least twice: at admission to the hospital and at discharge, then retested 3 months later in positive cases in order to be given the appropriate therapy.

## 1. Introduction

Antiphospholipid syndrome (APS) is an autoimmune disease characterized by the presence of antiphospholipid antibodies (aPLs) that target specific proteins in the blood and increase the risk of blood clots forming [1].

After the first publication of aPLs-positive tests in three COVID-19 patients in Wuhan, China, in January 2020 [2], there have been several subsequent reports of aPLs positivity in SARS-CoV-2-infected patients [3,4,5].

In APS, aPLs are usually associated with thromboses. Also, coagulopathy is one of the main mediators of morbidity and mortality caused by COVID-19, which increases the risk of arterial, venous, or microvascular thrombosis and, in severe forms of the disease, leads to pulmonary artery thromboembolism, deep vein thrombosis, myocardial infarction, and stroke [6]. Numerous pathophysiological mechanisms, such as epitheliopathy, cellular activation and inflammation, complement activation, autoantibody production, cytokine dysregulation, and fibrinolytic abnormalities, contribute to this hypercoagulable state. All these mechanisms support an immunothrombosis model, which is common in microvascular inflammatory diseases.

In COVID-19, immune effector cells secrete high levels of chemokines and proinflammatory cytokines (e.g., TGFβ, TNFα, IL-33, IL-18, IL-12, IFNγ, IL-6, IL-1β, and IFN-α) in response to SARS-CoV-2 infection [7]. According to some theories, the autoimmunity in COVID-19 can amplify the inflammatory pathways and make the disease worse [8]. Therefore, the prevention of a coronavirus immune evasion is crucial for maintaining the immune system by developing tailored and successful therapies [7].

It has been proven that severe COVID-19 and catastrophic APS (CAPS), the most severe variant of the APS spectrum with rapidly progressive thrombosis in multiple organs that can be fatal if not treated immediately, have been shown to share features, such as cytokine storm and multiorgan failure [9,10].

Since their initial discovery in patients with syphilis, the clinical significance of newly detected aPLs in the context of infections has attracted attention [11]. Many infections, like skin infections, human immunodeficiency virus (HIV) infections, hepatitis C virus, and urinary tract infections, have been shown to be associated with elevated aPLs [12,13,14]. Elevated aPLs have also been observed in patients vaccinated against COVID-19 [15].

The question therefore arises as to whether the elevated aPLs in COVID-19 are only due to infection or—since this disease has a prothrombotic potential—whether they may be due to APS triggered by the disease [16,17].

In addition to clinical criteria for thrombosis or pregnancy complications, for the diagnosis of APS, the detection of at least one of the APLs (lupus anticoagulant (LAC), anticardiolipin (aCL) antibodies or anti–beta2glycoprotein I (anti-β2GPI) antibodies of the IgG/IgM class) is mandatory, which must be confirmed as persistent at least twice, at least 12 weeks apart [18]. Recent studies have demonstrated the persistence of some APLs that occurred during COVID-19 in a follow-up, but their connection with APS has not been fully evaluated [19,20,21,22].

The aim of this study was to evaluate the new occurrence of APS in patients with severe COVID-19 hospitalized in the Intensive Care Unit due to pneumonia.

## 2. Materials and Methods

### 2.1. Patients and Data Extraction

**Figure 1:**
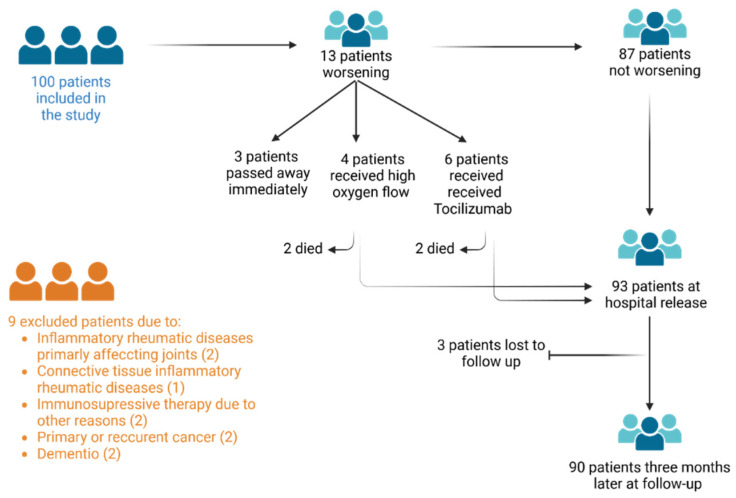
Study flow chart with the inclusion/exclusion criteria.

The study included patients with COVID-19 pneumonia admitted to the Intensive Care Unit (ICU) of the Clinical Hospital Center Bezanijska Kosa, Belgrade, and the General Hospital Pancevo, Serbia, between March 2021 and May 2021. All of them had positive SARS-CoV-2 RT-PCR results on nasopharyngeal swabs. Individuals with a history of APS and inflammatory rheumatic diseases, including primary joint diseases (rheumatoid arthritis and spondyloarthritis) and connective tissue diseases (systemic lupus erythematosus, systemic sclerosis, Sjögren’s disease, vasculitis, and dermatomyositis), individuals receiving immunosuppressive therapy, individuals with a history of cancer, those with HIV infection, and those with dementia were excluded (Figure 1).

All patients were treated according to the COVID-19 protocol with antibiotics, corticosteroids, anticoagulants, and special medications for concomitant diseases. In the ICU, patients were continuously supplied with oxygen and monitored with pulse oximetry and electrocardiography. Each patient’s body temperature, pulse, and blood pressure were also recorded every four hours.

Of the 100 patients enrolled, 13 deteriorated during their hospital stay, and 3 of them passed away shortly thereafter, making the laboratory tests impossible. Of the ten remaining patients, six were treated with tocilizumab, while four received high oxygen flow therapy. Despite the treatment, four more patients died. Ninety-three patients recovered and were discharged from hospital. Three months later, ninety people showed up for post/discharge follow-up.

Clinical criteria for APS, which included information on arterial/venous thrombosis and pregnancy complications, were collected during the patient’s hospitalization and from their personal history. Nine different types of aPLs were measured in the laboratory at four different time points, three times throughout the hospitalization: on admission, when the COVID-19 disease worsened, and when the patient was discharged from the hospital (initial testing) and once three months after discharge from the hospital (confirmatory test). Worsening was defined as the use of the anti-IL-6 drug tocilizumab, a cytokine storm, and the patient’s connection to a respirator.

The nine types of the collected aPLs were anticardiolipin [aCL] IgG/IgM/IgA, anti-β2-glycoproteinI [anti-β2GPI] IgG/IgM/IgA, and antiprothrombin-phosphatidilserine [aPS/PT] IgG/IgM/IgA antibodies. We could not employ the LA assay because we used anticoagulant drugs at therapeutic doses; the aPS/PT test was performed instead. To capture the majority of LA activity, antiprothrombin antibodies (aPS/PT) can be evaluated in conjunction with aCL and anti-β2GPI in patients in whom the LA test causes methodological problems, such as patients taking anticoagulant medications at therapeutic doses [23,24]. In addition, studies have shown a strong correlation between aPS/PT positivity and LA, with 77–100% of patients with isolated LA positivity also having positive aPS/PT [25].

Routine laboratory measurements were performed on all patients at the time of admission.

The Ethics Committees of Slovenia (#0120-7/2019/5, #0120-422/2020/6, and #0120-113/2021/4) and Serbia (132/2, 14 January 2021) approved the study. The study was conducted in accordance with the guidelines of the Declaration of Helsinki within the Serbian project of the Medical Faculty University of Belgrade, number 200100, and the Slovenian national research program number P3-0314. 

All enrolled patients have given their informed consent to participate.

### 2.2. Blood Sampling and Antiphospholipid Antibody Measurement

Samples were collected at four time points in accordance with established methodology for aPLs testing (aCL, anti-β2GPI, and aPS/PT of the IgG/IgM/IgA isotypes). After 30–60 min of clotting, the samples were centrifuged at 1500× *g* for 15 min (Heraeus Megafuge 16, ThermoFisher Scientific, Kalkberg, Osterode, Germany 2020) to separate the serum.

They were then filled into two Protein LoBind Tubes (Eppendorf) and stored at −80 °C. The samples were packed on dry ice to prevent evaporation before being delivered to the Department of Rheumatology at the University Medical Centre Ljubljana (UMCL), Slovenia, with the approval of the Ministry of Health of the Republic of Serbia. In accordance with previously published protocols and international recommendations [26,27,28], aPLs were measured using in-house enzyme-linked immunosorbent assays (ELISA): aCLs were assessed using a method first described in 1997 [26] and later evaluated [29]. Levels of anti-β2GPI were measured using the in-house ELISA [30] and evaluated by the European Forum for aPLs [31]. aPS/PT were assessed using an in-house ELISA, which was first described in 2011 [27] and has since been frequently evaluated [15,32]. 

The criteria for positive readings were values above the 99th percentile of healthy control, that is, for anti-β2GPI ≥ 2AU, aCL ≥ 11AU, and aPS/PT > 5AU.

### 2.3. Statistical Analyses

The data were described using frequencies and descriptive statistics (mean, standard deviation, median, and interquartile range). The one-sample Kolmogorov–Smirnov test was applied to determine whether the distribution of the data was normal. The paired samples T-test was used for comparisons of continuous variables that were normally distributed and the Mann–Whitney U test for those non-normally distributed. The Pearson Chi-Square test was employed to compare dichotomous scale variables, such as symptoms at admission, personal history, and aPL positivity between groups, followed by Fisher’s exact test when the predicted frequency of at least one cell was less than 5. Statistical significance was indicated by a *p*-value of 0.05 or less. All tests were performed using SPSS 21 (New York, NY, USA IBM Corp. Released 2021. IBM SPSS Statistics for Windows, Version 28.0. Armonk, NY, USA: IBM Corp).

## 3. Results

### 3.1. Patients’ Characteristics

Our cohort consisted of 65% men aged 27–78 years, with a mean (SD) age of 57.7 (13.2), and 35% women aged 32–77 years, with a mean (SD) age of 62.9 (9.5). The average length of hospitalization was 11.04 (5.03) days. 

The symptoms of COVID-19 on admission were fever (94%), dry cough (82%), productive cough (46%), dyspnea (55%), myalgia (70%), fatigue (88%), diarrhea (41%), headache (58%), and vomiting (22%).

### 3.2. Basic Laboratory Findings and Antiphospholipid Antibody Testing

In our group, 51% of patients had positive aPLs at at least one of the three initial assessments (hospital admission, exacerbation of COVID-19, and hospital discharge). Patients who tested positive for aPLs at one of the initial testings during their hospital stay did not differ from those who tested negative in terms of the erythrocyte sedimentation rate, C-reactive protein, lymphocytes, leucocytes, neutrophils, d-dimer, ferritin, troponin, procalcitonin and LDH levels, as well as red blood cell count, hemoglobin, hematocrit, and platelet count (Table 1).

Positive aPLs were found in 17.2% of patients at admission, 22.2% at exacerbation, 50% at hospital discharge, and 26.1% at follow-up. The main causes of aPLs positivity on admission were aCL IgG (59%), aCL IgM (23.5%), anti-β2GPI IgA (23.5%), and aPS/PT IgM (29.4%). 

At worsening, two patients had positive aPLs, both of which were in the aCL IgG class (100%), with one also having aPS/PT IgM. 

At hospital discharge, aPLs positivity was mostly caused by aCL IgG (59.6%), aCL IgM (46.8%) and anti-β2GPI IgG (14.9%). The most common aPLs at follow-up were aCL IgG (54.2%), aCL IgM (25%) and anti-β2GPI IgG (20.8%) (Table 2).

Also, no statistically significant difference was found between the transiently and persistently aPLs-positive patients in terms of the erythrocyte sedimentation rate, C-reactive protein, erythrocyte count, hemoglobin, hematocrit, lymphocytes, leukocytes, neutrophils, d-dimer, ferritin, troponin, procalcitonin, and LDH levels. 

### 3.3. COVID-19 Symptoms at Admission and Comorbidities with Regard to Antiphospholipid Antibody Positivity

Dyspnea, myalgia, and fatigue were significantly more common in patients who tested negative for aPLs at one of the initial tests (hospital admission, COVID-19 disease exacerbation, or hospital discharge) than in those who tested positive. However, these two groups did not differ in terms of fever, productive cough, dry cough, headache, diarrhea, or vomiting (Table 3).

In terms of comorbidities, there was no statistically significant difference between patients with negative and those with positive antiphospholipid antibodies in terms of hypertension, hyperlipidemia, diabetes mellitus, angina pectoris, and myocardial infarction in their personal history (Table 3).

### 3.4. Specific Subgroups of Patients

#### 3.4.1. Patients Who Developed Thrombosis While at the Hospital

Five of the six patients who developed thrombosis during hospitalization had pulmonary artery thrombosis, while one patient only had microthrombosis (Table 4). 

Four out of five patients with pulmonary artery thrombosis also had microthrombosis, one also had cerebral artery thrombosis, and one also had myocardial artery thrombosis. In 16,7% of patients, the intrahospital thrombosis was connected with aPLs. 

Of the three survivors who developed thrombosis during their hospital stay, only one (with pulmonary artery thrombosis, cerebral artery thrombosis, and microthrombosis) tested positive for aPLs: triple positive (for aCL IgG, anti-β2GPI IgM, and aPS/PT IgM) at admission, double (for aCL IgG and aPS/PT IgM) at discharge, and double (anti-β2GPI IgM and aPS/PT IgM) at follow-up (Table 4). 

None of the seven deceased patients tested positive for aPLs at the time of admission; at the time of deterioration, only one patient who died of pulmonary artery thrombosis and microthrombosis developed positive aCL IgG (Table 4).

Of the patients who passed away, three had pulmonary artery thrombosis, while the other four did not (two died of respiratory failure requiring respirator support, and two died of a cytokine storm and were treated with tocilizumab).

#### 3.4.2. Patients Who Had Thrombosis in Their Personal Anamnesis

Ten patients reported thrombosis in their personal anamnesis (PA), six had arterial thrombosis, and four had venous thrombosis; all of them tested negative for standard aPLs on admission (but one with lower extremity venous thrombosis tested for non-criteria positive anti-β2GPI IgA) (Table 5).

As for arterial thrombosis, five patients suffered from coronary artery thrombosis, and two suffered from cerebral artery thrombosis. Three of these patients tested newly positive aPLs at discharge; in one, the test was negative at follow-up, and the other two did not show for the follow-up. At follow-up three months after discharge, one patient with coronary artery thrombosis who had tested negative during hospitalization was found to be double positive for aCL IgM and anti-β2GPI IgM. However, he was not tracked for a further three months to confirm the presence of aPLs.

As for venous thrombosis, four patients had a history of deep vein thrombosis in the lower extremities, but only one of them tested consistently negative for APLs. One patient had positive APLs at discharge (double positive: anti-β2GPI IgA+ aCL IgG) and follow-up (single positive: aCL IgG) and was diagnosed with APS. Also, two patients were positive at discharge (both had double positivity for aCL IgM and aCL IgG), one of whom became negative, and one of whom did not return for follow-up (Table 5). 

#### 3.4.3. Patients with Pregnancy Complications

None of the women in our cohort were pregnant, and none became pregnant during their hospital stay or follow-up.

Two women had a history of abortions after the tenth week of gestation but did not test positive for aPLs at admission or follow-up, while one patient tested positive only at discharge. Our cohort did not include any women with a history of three or more abortions before the tenth week of gestation or who gave birth before the 34th week.

## 4. Discussion

Clinical and laboratory criteria for APS in patients hospitalized with COVID-19 pneumonia were thoroughly evaluated for the first time in this study. Clinical criteria included thrombosis occurring during hospitalization and thrombosis in personal history, as well as pregnancy complications during hospitalization and in personal history. Laboratory criteria included initial and confirmatory testing of aPLs during hospitalization and at a 3-month follow-up. As the associations between COVID-19 disease progression and aPLs have not yet been fully evaluated [5,6,7], initial aPLs testing was performed at three different time points throughout hospitalization: at hospital admission, at COVID-19 disease exacerbation, and at hospital discharge.

The lupus anticoagulant (LA) test was replaced by the aPS/PT in our study. LA should ideally be tested while patients are not taking anticoagulants [33]. Although low-molecular-weight heparin (LMWH) is not thought to interfere with the LA assessments when co-administered with neutralizators, this efficacy is limited to certain doses of LMWH, which are exclusively related to preventive doses (0.8–1.0 U/mL daily) [34,35]. When administered at higher and therapeutic doses (1.2–1.8 U/mL daily in our case), LMWH can lengthen major clotting tests and APTT durations depending on their anti-Fxa/FIIa ratio, which could compromise the detection of LA [33,35]. As a surrogate test for LA during anticoagulant therapy, some authors have suggest the use of anti-phosphatidyl-serine/prothrombin (aPS/PT) antibodies, as these are not affected by anticoagulants [36,37,38].

More than half of the patients in our cohort (51%) tested positive for aPLs at least once while in the hospital (hospital admission, deterioration, or discharge). Regarding aPLs, positivity upon admission was mostly due to aCL IgG (58.8%), aCL IgM (23.5%), anti-β2GPI IgA (23.5%), and aPS/PT IgM (29.4%). At release, positivity was mostly due to aCL IgG (59.6%), aCL IgM (46.8%), and—surprisingly, anti-β2GPI IgG—which was found to be newly positive in 12.8% of patients, who all tested negative at admission.

Of the ten patients whose COVID-19 worsened, two tested positive for aPLs at the time of worsening, and both showed aCL IgG positivity (100%). One of them died, while the other, who survived, was also positive for aPS/PT IgM.

The most common aPLs at follow-up were aCL IgG (54.2%), aCL IgM (25%), and anti-β2GPI IgG (20.8%).

In addition to the current study, the high incidence of aPLs in COVID-19 patients has been reported in numerous published papers, and the range of positivity for one of the aPLs was 5–71% [39]. This prevalence can vary considerably depending on the type of patient group (severe vs. non-severe patients) [40] and the investigated aPLs (consensus vs. supplementary criteria). In the absence of LA analysis, the most common aPLs are aCL [41,42,43] or anti-β2GPI [44]. The aCL IgG, aCL IgM, and anti-β2GPI positivity is around 15%. The prevalence of various extracriterial aPLs has been reported to be up to 24% for aPS/PT [45], 33% for aCL IgA [46], and 28.8% for anti-β2GPI IgA [47].

We verified that aPLs were not associated with a fatal outcome, as none of the patients who died in our study tested aPLs-positive upon hospital admission. As their condition worsened, only one of seven patients who died was found to be aPLs-positive, namely, for the aCL IgG class.

Furthermore, aPLs were also not associated with the occurrence of thrombosis in hospitals, as 83.3% of patients who suffered a thrombosis during hospitalization tested negative for aPLs upon admission.

According to one study, although 52.9% of COVID-19 patients were positive for at least one aPLs (29% LA-positive, 10.3% positive for 2 or more aPLs), they had no thrombotic events [12], which is consistent with our findings. The lack of correlation between the clinical signs of APS and aPLs positivity in COVID-19 patients was therefore attributed to the methodology of the different studies, as most of them did not include control cohorts, so there was no population to compare with COVID-19. Studies with control groups showed no significant differences in the prevalence of aPLs in COVID-19 compared to other infections or autoimmune diseases [46,48,49,50], with the exception of IgG and IgM aCL (59% versus 35% and 32% versus 10%, respectively) [41], LA [51], and IgA aβ2GPI [52]. However, in most studies published to date, only a single measurement point for aPLs was recorded—usually during the COVID-19 exacerbation—without follow-up and confirmation after three months, as required by the laboratory criteria for antiphospholipid syndrome.

Our findings are supported by the Japanese COVID-19 patient population, in which aPLs are also frequently detected, but their titer and prevalence had no impact on thrombotic events [53]. In addition, Zekic et al. reported no association between the presence of aPLs in severe COVID-19 and thrombosis or worse outcomes [22]. However, it is a fact that the common protein signature of COVID-19 patients with comorbidities revealed alterations in acute-phase response proteins, coagulation and complement pathways, tissue injury and remodeling, and cholesterol metabolism [54].

Due to molecular mimicry mechanisms, aPLs can also occur in patients with other important and diverse infections/diseases (HIV, hepatitis C, parvovirus B19, etc.) [55].

In bacterial infections—especially sepsis—the acute-phase response may be associated with the presence of aPLs, but these are usually only transiently raised and not associated with the presence of autoimmune disease. When the infection resolves, aPLs return to normal levels in most patients [56,57,58]. In bacterial and viral infections, such as influenza, aPLs consist mainly of the LA and aCL types; their incidence is often much lower than in severe COVID-19, and the thrombotic effects in these infections are less frequent. Hepatitis, HIV, and Epstein–Barr virus can trigger aPLs, especially in the setting of chronic or severe disease, but their incidence and duration are often much lower than in severe COVID-19 [59].

Persistent aPLs are observed in certain chronic infections, particularly HIV (49.75%), hepatitis B virus (HBV) (24%), and hepatitis C virus (HCV) (20%). However, they are extremely rarely presented with anti-β2GPI antibodies and usually do not correlate with the risk of thrombosis [59,60,61].

In cases where thromboembolic events occurred, the most commonly reported viruses were hepatitis C virus (HCV) and human immunodeficiency virus (HIV). The most common cause of antibodies without thromboembolic events was parvovirus B19 [61].

When comparing patients with infections to those with APS, the profile of aPLs differs [12], and recent studies have shown that COVID-19 patients also show different aPLs profiles than APS but similar to those of other infections [12].

The major immunogenic epitope in APS patients that is closely associated with thrombosis is the β2GPI domain [62], but only 5% of COVID-19 patients with aPLs positivity had the β2GPI I domain [44].

When assessed at a single time point, the prevalence and titers of aPLs or LA in COVID-19 were neither consistently elevated nor linked with thrombosis, as shown in a multicenter analysis [49].

Also, in our study, the majority of aPLs consisted of aCL IgG and aCL IgM at hospitalization. As the disease resolved, anti-β2GPI IgG, which is mostly associated with thrombosis in APS, became newly positive after initially testing negative at hospital admission. This new-positive anti-β2GPI IgG at hospital discharge—when COVID-19 disease went into remission—was confirmed 3 months later at follow-up. This positivity was not related to thrombosis during hospitalization and personal history; however, these patients were not further evaluated for thrombosis in their lifetime.

The prevalence of non-criteria aPS/PT in our study was 23.5% at admission and 12.8% at discharge.

In patients hospitalized with COVID-19 pneumonia, we found an APS incidence rate of 2 per 100 people, which means that two patients received a new APS diagnosis. One of the patients who obtained pulmonary artery thrombosis with microthrombosis and cerebral artery thrombosis during hospitalization, aCL IgG, anti-β2GPI IgM, and aPS/PT IgM were triple aPLs-positive at admission; aCL IgG and aPS/PT IgM were double positive at discharge; and anti-β2GPI IgM and aPS/PT IgM were double positive at follow-up. The other patient had a history of deep vein thrombosis and tested positive for anti-β2GPI IgA on admission, anti-β2GPI IgA and aCL IgG on discharge, and aCL IgG on follow-up.

The incidence of confirmed APS in our cohort is 2%. In the general population, the estimated incidence of APS is between 1 and 2 cases per 100,000, with a lower incidence in Europe [63,64], so we found a thousand-fold higher incidence in our group.

In our study, there were four additional patients with intrahospital thrombosis or a history of thrombosis who tested positive for aPLs during hospitalization but did not attend the follow-up or had passed away. For this reason, their status as APS could not be confirmed. However, if they had attended, the incidence may have been even higher.

All four of these individuals had negative aPLs testing upon admission to the hospital, and most of them went on to test positive upon discharge as they recovered. Two of them had coronary artery thrombosis in their personal anamnesis (PA) and were newly aPLs-positive at discharge (one double positive for aCL IgM and aCL IgA, the other for aCL IgG only), but they did not show up for follow-up. One of these patients had deep vein thrombosis of the lower extremities in his PA and tested double positive (aCL IgG, aCL IgM) at hospital discharge but did not attend the follow-up visit. One patient developed pulmonary artery thrombosis and microthrombosis (with aCL IgG positivity) during hospitalization but could not be followed up as he died.

In total, there were six patients in our study with a history of thrombosis who tested aPLs-negative upon admission and transitioned to aPLs-positive upon discharge: three of them with arterial thrombosis (one with cerebral artery thrombosis and two with coronary artery thrombosis) and three with deep vein thrombosis.

In addition, there was one patient with coronary artery thrombosis who tested negative at all three time points during hospitalization but reversed to double positive for aCL IgM and anti-β2GPI IgM at the follow-up three months later. However, this patient was not further tracked for the confirmation of aPLs.

### 4.1. COVID-19: The Optimal Anticoagulation Therapy for Arterial/Venous Thrombosis and aPLs

Patients hospitalized for severe COVID-19 and receiving therapeutic doses of LMWH should be tested for antiphospholipid antibodies [aPLs]: anticardiolipin [aCL] IgG/IgM, anti-β2-glycoproteinI [anti-β2GPI] IgG/IgM, and antiprothrombin-phosphatidilserine antibody [aPS/PT] IgG/IgM at least twice: when admitted and when discharged. If at least one of the aPLs tested positive on one occasion, the test should be repeated after three months. 

Patients with a history of severe COVID-19 (vascular, pulmonary, or neurological manifestations) and those with post-COVID syndrome should be tested once for aCL IgG/IgM, anti-β2GPI IgG/IgM, and lupus anticoagulant [LA], and retested after three months if at least one of the aPLs was positive.

If the aPLs are positive twice at least three months apart, individuals should be screened for current and previous thrombosis; if arterial or venous thrombosis is detected at that time or in the patient’s personal history, the patient should receive lifelong anticoagulation therapy as recommended. The recommendation for aPLs testing assessment, evaluation of thrombosis, and optimal anticoagulation therapy is as follows.

#### 4.1.1. Venous Thrombosis 

**Figure 2:**
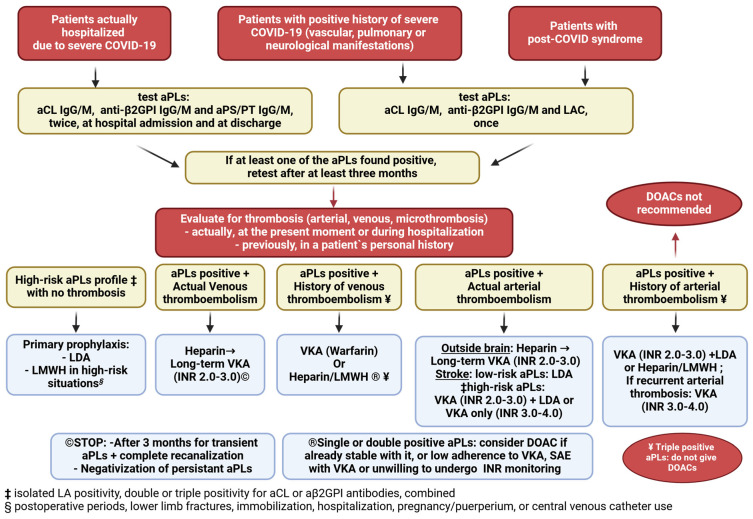
Recommendations for the testing of current or previous COVID-19 patients for antiphospholipid antibodies and for their evaluation with regard to current/past thrombosis with the proposed therapy in positive cases. aPLs, antiphospholipid antibodies; aCL, anticardiolipin antibodies; anti-β2GPI, anti-β2-glycoprotein I antibodies; aPS/PT, anti-phosphatidylserine-prothrombin antibodies; LDA, low-dose aspirin; LMWH, low molecular-weight heparin (Fraxiparine); VKA, vitamin K antagonists (warfarin); DOAC (s), direct oral anticoagulants.

If a patient with venous thrombosis is treated with therapeutic doses of LMWH and tests positive for aCL IgG/IgM, anti-β2GPI IgG/IgM, or aPS/PT IgG/IgM, or if he has a personal history of venous thrombosis and tests positive for aCL IgG/IgM, anti-β2GPI IgG/IgM, or LA and tests positive again after at least three months, the recommendation is as described below (Figure 2). 

In patients with actual venous thrombosis who are aPLs-positive, heparin is administered, followed by long-term vitamin K antagonists—VKA (warfarin) (INR 2.0–3.0). Discontinue treatment if persistent aPLs become negative over time.

For APS patients with a personal history of deep vein thrombosis and a confirmed aPLs profile, treatment with VKA (Warfarin) or low molecular weight heparin—LMWH (Fraxiparine)—is recommended. According to EULAR, ASH, and ISTH recommendations, direct anticoagulants (DOACs) may be considered if a patient is already stable on DOACs, has poor adherence to VKA or is reluctant to undergo INR monitoring, has a severe allergy, or experiences additional side effects from VKA [65]. Due to their embryotoxic effects, VKAs are prohibited during pregnancy. Instead, LMWH should be used in therapeutic doses regularly adjusted to body weight if venous thrombosis occurs during pregnancy.

#### 4.1.2. Arterial Thrombosis

If a patient has tested positive for aCL IgG/IgM, anti-β2GPI I IgG/IgM, or LA or is receiving a therapeutic dose of LMWH and has tested positive for aCL IgG/IgM, anti-β2GPI IgG/IgM, or aPS/PT IgG/IgM and tests positive again after at least three months and has arterial thrombosis, the recommendation is as shown in Figure 2.

If a patient with proven aPLs has a recent arterial thrombosis outside the cerebral circulation, heparin is administered, followed by long-term treatment with vitamin K antagonists—VKA (warfarin)—with a target INR 2.0 to 3.0, just as in the case of venous thrombosis [65].

On the other hand, when arterial thrombosis leads to stroke, the question of whether the aPLs have a low-risk or high-risk profile is of primary concern. A low-risk profile of aPLs is indicated by isolated positivity for aCL or anti-β2GPI antibodies, whereas a high-risk profile is determined by isolated LA positivity, double or triple positivity for aCL or anti-β2GPI, combined.

In cases with stroke and a low-risk aPLs profile, low-dose aspirin (LDA) is recommended.

In cases with cerebral thrombosis and a high-risk aPLs profile, as was the case in one of the newly diagnosed APS patients in our cohort who suffered pulmonary artery thrombosis with microthrombosis and cerebral artery thrombosis during hospitalization and was double aPLs-positive at discharge and follow-up, the best treatment option would be VKA (warfarin) (INR 2.0–3.0) in combination with low-dose aspirin (LDA) or VKA alone (INR 3.0–4.0), especially in cases with cardiovascular risk factors and progression, recurrent thrombosis on VKA (INR 2.0–3.0), and a low risk of bleeding [66].

In patients without current or previous thrombosis or obstetric problems but with a high-risk aPLs profile (defined as isolated LA positivity, double or triple positivity for aCL, or anti-β2GPI antibodies, combined), LDA prophylaxis is suggested, albeit with little evidence. It is generally accepted that LMWH should be used in these patients, as in all other severe thrombophilia abnormalities, in high-risk situations, such as postoperative periods, lower limb fractures, immobilization, hospitalization, pregnancy/postpartum, or central venous catheter placement [66] (Figure 2).

Statins, as lipid-modifying drugs, can be used in APS, even in the absence of significant dyslipidemia, to improve the stability of atherosclerotic plaques and reduce oxidative stress and inflammation [67]. In patients with coronary artery disease, statins can also reduce aPLs titers [68]. Statin use, in addition to usual therapy, was associated with a lower incidence of recurrent thrombosis in a multivariable regression analysis of 184 individuals with APS [69]. Even after controlling for vascular risk factors, the type of conventional therapy, and aPLs profile, this association remained significant. If there are no contraindications, all APS patients are therefore advised to take statins.

The shift from negativity of aPLs to positivity throughout the COVID-19 recovery phase in our study is difficult to explain, as it has not been described in other infections and in other studies. On the contrary, the switch from aPLs positivity to negativity is usually observed in the recovery phase of infections.

In autoimmune diseases, the presence of aPLs is often constant, but a switch from positive to negative aPLs has been documented after thrombosis. For example, the complete loss of antiphospholipid positivity after thrombosis has been described in SLE, with losses of up to 41% for aCL IgG, 51% for IgM, and 50% for IgA, but only 20% for lupus anticoagulant [70].

According to the literature, clinically visible thrombosis in COVID-19 is not clearly associated with aPLs, as most studies showed no association between aPLs and the severity of COVID-19, thrombosis, or other APS-related symptoms [12,41,42,44,49,71,72,73,74,75,76]. Some authors suggested that the aPLs detected in COVID-19 are different from those observed in APS patients, implying that it is an epiphenomenon without pathogenicity [44]. Positive aPLs are found in up to 54.8% of patients with COVID-19, with more than half of patients carrying aCL IgG. Even in an expanded panel of aPLs, only aCL IgG was associated with the severity of COVID-19 [77]. However, some studies have found a higher prevalence of aPLs in critically ill patients admitted to the ICU with high mortality, ARDS, and renal or ventilatory failure [43,47,48,52,72,78,79,80]. Fewer studies have found an association between aPLs and thrombotic events, including stroke [20,21,46,47,81,82].

Thrombosis in the post-acute phase (PAP) of COVID-19, also known as post-COVID syndrome (PCS), has been described in some studies. In a prospective analysis of 361 patients, an association was found between aPLs and the occurrence of thrombosis in the first six months after COVID-19 (OR: 3.7, 95% CI (1.7–8.1) [46].

In patients with the PAP phase of COVID-19, an increase in proinflammatory cytokines, namely, IFN-α, TNF-α, G-CSF, IL17A, IL-6, IL1-β, and IL-13, has been confirmed in a number of studies [83,84,85]. In addition, a significant decrease in IL-10 was also found [83].

Bertin et al. discovered that chronic aCL positivity could be a biological indicator of post-COVID syndrome (PCS) [86]. PCS could be explained by subclinical micro-thrombotic events caused by the presence of aPLs, suggesting that endothelial tissue remains damaged even after patients recover from the acute clinical phase of the disease. Moreover, in some cases, it could be reactivated in different regions of the microvasculature, such as the vasa vasorum of the peripheral nerves and the cerebral microvasculature, leading to a chronic proinflammatory state (endothelitis) that may cause chronic neuropathic or memory changes [55,87].

A significant proportion of severe and PAP COVID-19 patients exhibited aCL IgM/IgG and anti-B2GPI IgM/IgG positivity, with persistent low-level pro-thrombotic potential activity, highlighted by a sustained imbalance in the innate immune response and persistence of NETs markers [88]. Although data suggest that persistent autoantibodies may be associated with post-COVID syndrome, their existence is thought to have limited clinical significance [88].

Breaking this down, the current study has revealed a connection between the presence of aPLs in subsiding disease and thrombosis in personal history, which may be of crucial importance for personalized diagnostic and therapeutic methods to improve patient outcomes.

## 5. Conclusions

The novelty of this study is that some of the severe COVID-19 patients went from negativity of aPLs upon admission to positivity on recovery—when discharged from the hospital, as COVID-19 disease went into remission. In conjunction with thrombosis, 60% of patients with a personal history of arterial or venous thromboembolism, which is not typically assessed in COVID-19, were tested newly positive for aPLs at hospital discharge. On the other hand, only 16.7% of patients who developed thrombosis during hospitalization tested positive for aPLs on admission, and none of them tested newly positive for aPLs at discharge.

Also, anti-β2GPI IgG, which is otherwise connected with thrombosis, tested newly positive in 14.9% and 20.8% of aPLs-positive patients at discharge and was confirmed at a 3-month follow-up, but these patients were not followed further for thrombosis in their lifetime.

The results of the current study emphasize the need to monitor aPLs in all COVID-19 patients, as well as in those in the post-acute phase; link them to data on recent thrombosis, as recommended by other authors [89]; and monitor for thrombosis in the lifetime history, as recommended in the current study.

The main limitation of our study is that we could not perform the LA measurement because all our patients were already receiving anticoagulation treatment with LMWH at therapeutic doses for COVID-19 pneumonia. Although LMWH is not thought to alter the LA test when administered with neutralizers, it is only effective at certain, typically preventive, and low doses of LMWH (0.8–1.0 U/mL). We used therapeutic doses of LMWH in COVID-19 pneumonia. In this case, anticoagulants may interfere with LA detection tests, sometimes resulting in false-positive or false-negative LA.

### What Should Be Done if a Patient Has Severe COVID-19, a History of Severe COVID-19 or Post-COVID Syndrome?

It is recommended that all patients admitted to the hospital with COVID-19 are screened for thrombosis during hospitalization and in personal history and tested for aPLs at least twice: when admitted to the hospital and when discharged. Given the high incidence of APS demonstrated in this study, individuals who test positive at least once during hospitalization should be retested at a follow-up visit at least three months later for confirmation.

Patients who have previously had severe COVID-19 with vascular/neurological/pulmonary manifestations and patients with long-COVID or post-COVID syndrome should be screened for current/past thrombosis and tested for aPLs, with positive cases retested three months later.

If an APS diagnosis is confirmed, the patient should receive lifelong anticoagulation therapy in accordance with established protocols. This practice may contribute to the early recognition and management of potential complications associated with antiphospholipid syndrome.

## Figures and Tables

**Table 1:** Laboratory parameters in antiphospholipid antibodies-positive and negative patients * upon admission to the hospital.

Mean ± SD/Median (IQR) ^†^	Normal Range	aPLs-Positive n = 51	aPLs-Negative n = 49	*p*-Value *t* test
Erythrocyte sedimentation rate (mm/h)	0–15	54.98 (23.04)	46.53 (21.26)	0.060
C- reactive protein (mg/L) ^†^	<5.0	112.12 (71.94)	100.08 (71.72)	0.404
Erytrocytes (×10^12^/L)	4.50–6.30	4.41 (0.52)	4.58 (0.55)	0.129
Hemoglobin (g/L)	140–175	129.99 (15.96)	133.92 (14.41)	0.198
Hematocrit (%)	0.400–0.520	0.396 (0.045)	0.403 (0.043)	0.426
Leucocytes (×10^9^/L)	4.40–11.50	5.95 (2.37)	6.21 (2.62)	0.126
Neutrophils (×10^9^/L)	2.20–8.05	4.33 (2.33)	4.94 (2.42)	0.206
Lymphocytes (×10^9^/L)	1.10–4.60	1.01 (0.55)	0.85 (0.41)	0.152 ‡
Platelets (×10^9^/L)	150–400	229.51 (78.42)	210.10 (78.84)	0.220
D-dimer (mg/L) ^†^	<0.5	1.34 (3.12)/0.63 (0.85)	1.43 (3.36)/ 0.77 (0.94)	0.183 ‡
Ferritin (µg/L)	4.63–204.00	1069.57 (689.34)	998.63 (676.33)	0.516 ‡
Troponin (ng/L) ^†^	0–11.6	21.87 (65.09)/ 5.92 (7.04)	28.18 (114.80) 5.87 (7.92)	0.842 ‡
Procalcitonin (ng/mL) ^†^	<0.08	0.094 (0.082)/0.070 (0.08)	0.509 (2.693) 0.080 (0.08)	0.896 ‡
LDH (U/L)	230–480	668.84 (238.38)	653.69 (241.47)	0.753

* Positive for at least one of nine types of antiphospholipid antibodies (aPLs): anticardiolipin [CL], anti-β2 glycoprotein I [anti-β2GPI] and antiprothrombin-phosphatidilserin [aPS/PT] antibodies of the IgG or IgM or IgA class determined at least once during hospitalization (at admission, upon exacerbation of COVID-19 disease, or at discharge); n, number of patients. ^†^ For data sets with outliers, results are given as median (IQR); IQR, interquartile range. ‡ Mann–Whitney U test.

**Table 2:** Antiphospholipid antibody positivity in COVID-19 patients admitted to ICU for pneumonia: at hospital admission, during COVID-19 disease exacerbation, at discharge, and at 3-month follow-up.

	All Patients				aPLs-Positive Patients			
Type of aPLs No (%)	Admission (n = 100)	Worsening (n = 9)	Disharge (n = 93)	Follow-Up (n = 90)	Admission (n = 17)	Worsening (n = 2)	Discharge (n = 47)	Follow-Up (n = 24)
aCL IgG	10 (10.0)	2 (22.2)	28 (30.1)	13 (14.4)	10 (58.8)	2 (100.0)	28 (59.6)	13 (54.2)
aCL IgM	4 (4.0)	0 (0.0)	22 (23.7)	6 (6.7)	4 (23.5)	0 (0.0)	22 (46.8)	6 (25.0)
aCL IgA	3 (3.0)	0 (0.0)	1 (1.1)	0 (0.0)	3 (17.6)	0 (0.0)	1 (2.1)	0 (0.0)
anti-β2GPI IgG	0 (0.0)	0 (0.0)	7 (7.5)	5 (5.6)	0 (0.0)	0 (0.0)	7 (14.9)	5 (20.8)
anti-β2GPI IgM	1 (1.0)	0 (0.0)	2 (2.2)	4 (4.4)	1 (5.9)	0 (0.0)	2 (4.3)	4 (16.7)
anti-β2GPI IgA	4 (4.0)	0 (0.0)	4 (4.3)	4 (4.4)	4 (23.5)	0 (0.0)	4 (8.5)	4 (16.7)
aPS/PT IgG	0 (0.0)	0 (0.0)	0 (0.0)	0 (0.0)	0 (0.0)	0 (0.0)	0 (0.0)	0 (0.0)
aPS/PT IgM	5 (5.1)	1 (11.1)	5 (5.4)	4 (4.4)	5 (29.4)	1 (50.0)	5 (10.6)	4 (16.7)
aPS/PT IgA	1 (1.0)	0 (0.0)	2 (2.2)	1 (1.1)	1 (5.9)	0 (0.0)	2 (4.3)	1 (4.2)
aPLs positivity (9) ^1^	17 (17.0)	2 (22.2)	46 (50.0)	23 (26.1)	/	/	/	/
aCL positivity (3) ^2^	13 (13.0)	2 (22.2)	41 (44.1)	18 (20.0)	13 (76.5)	2 (100.0)	41 (87.2)	18 (75.0)
anti-β2GPI positivity (3) ^3^	5 (5.0)	0 (0.0)	12 (12.9)	11 (12.2)	5 (29.4)	0 (0.0)	11 (23.4)	11 (45.8)
aPS/PT positivity (3) ^4^	4 (4.0)	1 (11.1)	6 (6.5)	4 (4.4)	4 (23.5)	1 (50.0)	6 (12.8)	4 (16.7)
Double positivity ^5^	1 (1.0)	1 (11.1)	9 (9.7)	5 (5.6)	1 (5.9)	1 (50.0)	9 (19.1)	5 (20.8)
Triple positivity ^6^	2 (2.0)	/	1 (1.1)	2 (2.2)	2 (11.8)	/	1 (2.1)	2 (8.3)

Note: Positive values were defined as those above the 99th percentile of the healthy control population; No (%), number (percent); n, number of patients; aPLs, antiphospholipid antibodies; aCL, anticardiolipin antibodies; anti-β2GPI, anti-β2-glycoprotein I antibodies; aPS/PT, anti-phosphatidylserine-prothrombin antibodies; ^1^ aPLs positivity, at least 1 of 9 evaluated antiphospholipid antibodies (aCL, anti-β2GPI, aPS/PT of class IgG, IgM, or IgA) above the reference range; ^2^ aCL positivity, at least 1 of 3 aCL above the reference range (IgG, IgM, or IgA); ^3^ anti-β2GPI positivity, at least 1 of 3 anti-β2GPI above the reference range (IgG, IgM, or IgA); ^4^ aPS/PT positivity, at least 1 of 3 aPS/PT above the reference range (IgG, IgM, or IgA); ^5^ at least 1 positive in 2 of 3 classes of aPLs: aCL, anti-β2GPI, aPS/PT; ^6^ at least 1 in 3 of 3 classes of aPLs positive: aCL, anti-β2GPI, or aPS/PT.

**Table 3:** Antiphospholipid antibody ^§^-positive and -negative patients according to occurrence of thrombosis, pregnancy complications, COVID-19 symptoms at admission, and comorbidities.

	Patient Characteristics No (%)	aPLs-Positive n = 51	aPLs-Negative n = 49	*p*-Value Chi-Square
THROBOSIS DURING HOSPITALIZATION AND IN PERSONAL HISTORY; PREGNANCY COMPLICATIONS	Age (mean SD)	61.37 (11.58)	57.67 (12.77)	0.132
	Hospitalization (days)	11.53 (5.24)	10.49 (4.77)	0.324
	Thrombosis during hospitalization	2 (3.9)	4 (8.2)	0.432 ‡‡
	During hospitalization pulmonary artery thrombosis	2 (3.9)	3 (6.1)	0.675 ‡‡
	During hospitalization arterial thrombosis	1 (2.0)	1 (2.1)	1.000 ‡‡
	During hospitalization microthrombosis	2 (3.9)	3 (6.1)	0.675 ‡‡
	Thrombosis in personal history	6 (11.8)	4 (8.2)	0.741 ‡‡
	Thrombosis during hospitalization and in personal history	8 (15.7)	7 (14.3)	0.845
	One or more abortions after X week of gestation	1 (2.0)	1 (2.1)	1.000 ‡‡
COVID-19 SYPTOMS AT ADMISSION	Fever	46 (90.2)	48 (98.0)	0.102
	Dry cough	40 (78.4)	42 (85.7)	0.343
	Productive cough	23 (45.1)	23 (46.9)	0.854
	Dyspnea	23 (45.1)	32 (65.3)	0.042 *
	Myalgia	30 (58.8)	40 (81.6)	0.013 *
	Fatigue	39 (76.5)	46 (93.9)	0.046 *
	Diarrhea	19 (37.3)	22 (44.9)	0.437
	Headache	31 (60.8)	27 (55.1)	0.565
	Vomiting	10 (19.6)	12 (24.5)	0.556
PERSONAL ANAMNESIS FOR COMORBIDITIES	Hypertension in personal anamnesis	27 (52.9)	23 (46.9)	0.548
	Hyperlipidemia in personal anamnesis	23 (45.1)	21 (43.8)	0.893
	Diabetes mellitus in personal anamnesis	15 (29.4)	10 (20.8)	0.326
	Angina pectoris in personal anamnesis	6 (11.8)	6 (12.2)	0.941
	Infarctus myocardii in personal anamnesis	2 (3.9)	3 (6.1)	0.481 ‡‡
	PAH in personal anamnesis	1 (2.0)	1 (2.1)	0.966 ‡‡
	Pneumonia in personal anamnesis	17 (33.3)	13 (26.5)	0.458

No (%), number (percent); n, number of patients. ^§^ Positive for at least one out of nine types of antiphospholipid antibodies (aPLs): anticardiolipin [aCL], anti-β2-glycoproteinI [anti-β2GPI] and antiprothrombin-phosphatidilserin antibodies [aPS/PT], all of the IgG or IgM or IgA class during hospitalization (at hospital admission, at deterioration of the COVID-19 disease, or at discharge from the hospital), PAH, pulmonary artery hypertension; * *p* < 0.05, ‡‡ Fisher’s exact test.

**Table 4:** Intrahospital thrombosis in patients hospitalized with COVID-19 pneumonia in the intensive care unit in terms of survival and antiphospholipid antibody profile at admission, exacerbation, hospital discharge, and at three-month follow-up.

		Patients Who Died											
Pt No.	Thrombosis Emerged During Hospitalization	Admission			Worsening			Discharge			Follow-Up		
		aCL	anti -β2GPI	aPS/PT	aCL	anti -β2GPI	aPS/PT	aCL	anti -β2GPI	aPS/PT	aCL	anti -β2GPI	aPS/PT
		IgG/IgM/IgA	IgG/IgM/IgA	IgG/IgM/IgA	IgG/IgM/IgA	IgG/IgM/IgA	IgG/IgM/IgA	IgG/IgM/IgA	IgG/IgM/IgA	IgG/IgM/IgA	IgG/IgM/IgA	IgG/IgM/IgA	IgG/IgM/IgA
5	No thrombosis	0/0/0	0/0/0	0/0/0	0/0/0	0/0/0	0/0/0	/	/	/	/	/	/
17	PA, microthrombosis, Myocardial Infarction	0/0/0	0/0/0	0/0/0	/	/	/	/	/	/	/	/	/
40	No thrombosis	0/0/0	0/0/0	0/0/0	0/0/0	0/0/0	0/0/0	/	/	/	/	/	/
64	No thrombosis	0/0/0	0/0/0	0/0/0	0/0/0	0/0/0	0/0/0	/	/	/	/	/	/
70	PA, microthrombosis,	0/0/0	0/0/0	0/0/0	1/0/0	0/0/0	0/0/0	/	/	/	/	/	/
71	No thrombosis	0/0/0	0/0/0	0/0/0	0/0/0	0/0/0	0/0/0	/	/	/	/	/	/
92	PA	0/0/0	0/0/0	0/0/0	/	/	/	/	/	/	/	/	/
		**Patients Who Survived**											
49	PA, Microthrombosis, Cerebral art thrombosis	1/0/0	0/1/0	0/1/0	1/0/0	0/0/0	0/1/0	1/0/0	0/0/0	0/1/0	0/0/0	0/1/0	0/1/0
85	PA, Microthrombosis	0/0/0	0/0/0	0/0/0	0/0/0	0/0/0	0/0/0	0/0/0	0/0/0	0/0/0	0/0/0	0/0/0	0/0/0
91	Microthrombosis	0/0/0	0/0/0	0/0/0	/	/	/	0/0/0	0/0/0	0/0/0	0/0/0	0/0/0	0/0/0

Cohort consists of 100 patients; Pt No., patient number; aPLs, antiphospholipid antibodies; aCL, anticardiolipin antibodies; anti -β2GPI, anti-β2-glycoprotein I antibodies; aPS/PT, anti-phosphatidylserine-prothrombin antibodies; PA, pulmonary artery thrombosis.

**Table 5:** Personal history of thrombosis and pregnancy complications in patients hospitalized with COVID-19 pneumonia in the ICU in terms of survival and their antiphospholipid antibody profile at the time of admission, deterioration, hospital discharge, and three-month follow-up *.

		Arterial Thrombosis and Venous Thrombosis in Personal History											
Pt No.		Admission			Worsening			Discharge			Follow-Up		
	aPLs	aCL	anti -β2GPI	aPS/PT	aCL	anti -β2GPI	aPS/PT	aCL	anti -β2GPI	aPS/PT	aCL	anti -β2GPI	aPS/PT
	Type of aPLs	IgG/IgM/IgA	IgG/IgM/IgA	IgG/IgM/IgA	IgG/IgM/IgA	IgG/IgM/IgA	IgG/IgM/IgA	IgG/IgM/IgA	IgG/IgM/IgA	IgG/IgM/IgA	IgG/IgM/IgA	IgG/IgM/IgA	IgG/IgM/IgA
	**Localization**	**Arterial Thrombosis in Personal History**											
15	Cerebral artery	0/0/0	0/0/0	0/0/0	/	/	/	0/1/0	0/0/0	0/0/0	0/0/0	0/0/0	0/0/0
18	Coronary artery	0/0/0	0/0/0	0/0/0	/	/	/	0/0/0	0/0/0	0/0/0	0/1/0	0/1/0	0/0/0
21	Coronary artery	0/0/0	0/0/0	0/0/0	/	/	/	0/0/0	0/0/0	0/0/0	0/0/0	0/0/0	0/0/0
26	Coronary artery	0/0/0	0/0/0	0/0/0	0/0/0	0/0/0	0/0/0	0/1/1	0/0/0	0/0/0	/	/	/
31	Coronary artery	0/0/0	0/0/0	0/0/0	/	/	/	1/0/0	0/0/0	0/0/0	/	/	/
86	Cerebral artery, Coronary artery	0/0/0	0/0/0	0/0/0	/	/	/	0/0/0	0/0/0	0/0/0	0/0/0	0/0/0	0/0/0
	**Localization**	**Venous Thrombosis in Personal History**											
22	Deep vein thrombosis LE	0/0/0	0/0/1	0/0/0	/	/	/	1/0/0	0/0/1	0/0/0	1/0/0	0/0/0	0/0/0
37	Deep vein thrombosis LE	0/0/0	0/0/0	0/0/0	/	/	/	1/1/0	0/0/0	0/0/0	0/0/0	0/0/0	0/0/0
56	Deep vein thrombosis LE	0/0/0	0/0/0	0/0/0	/	/	/	1/1/0	0/0/0	0/0/0	/	/	/
94	Deep vein thrombosis LE	0/0/0	0/0/0	0/0/0	/	/	/	0/0/0	0/0/0	0/0/0	0/0/0	0/0/0	0/0/0
		**Pregnancy Complications in Personal History**											
8	Abortion after X weeks	0/0/0	0/0/0	0/0/0	/	/	/	0/1/0	0/0/0	0/0/0	0/0/0	0/0/0	0/0/0
54	Abortion after X weeks	0/0/0	0/0/0	0/0/0	/	/	/	0/0/0	0/0/0	0/0/0	0/0/0	0/0/0	0/0/0

* Cohort consists of 100 patients; Pt No., patient number; aPLs, antiphospholipid antibodies; aCL, anticardiolipin antibodies; anti -β2GPI, anti-β2-glycoprotein I antibodies; aPS/PT, anti-phosphatidylserine-prothrombin antibodies; deep vein thrombosis LE, deep vein thrombosis of the lower extremity; abortion after X weeks, abortion after X weeks of gestation.

## Data Availability

The data presented in this study are available upon request from the corresponding author. The data are not publicly available due to ethical reasons.
